# Development and Characterization of an Injectable Alginate/Chitosan Composite Hydrogel Reinforced with Cyclic-RGD Functionalized Graphene Oxide for Potential Tissue Regeneration Applications

**DOI:** 10.3390/ph18050616

**Published:** 2025-04-23

**Authors:** Mildred A. Sauce-Guevara, Sergio D. García-Schejtman, Emilio I. Alarcon, Sergio A. Bernal-Chavez, Miguel A. Mendez-Rojas

**Affiliations:** 1 Department of Chemical and Biological Sciences, Universidad de las Americas Puebla, Ex-Hacienda de Santa Catarina Martir s/n, San Andres Cholula, Puebla 72820, Mexico; mildred.saucega@udlap.mx; 2 Bioengineering and Therapeutic Solutions (BEaTS) Program, University of Ottawa Heart Institute, Ottawa, ON K1Y 4W7, Canada; dgarcia@unc.edu.ar (S.D.G.-S.); ealarcon@uottawa.ca (E.I.A.); 3 Department of Biochemistry, Microbiology, and Immunology, University of Ottawa, Ottawa, ON K1H 8M5, Canada

**Keywords:** tissue regeneration, hydrogel composite, alginate, chitosan, graphene oxide, injectability

## Abstract

**Background:** In tissue engineering, developing injectable hydrogels with tailored mechanical and bioactive properties remains a challenge. This study introduces an injectable hydrogel composite for soft tissue regeneration, composed of oxidized alginate (OA) and N-succinyl chitosan (NSC) cross-linked via Schiff base reaction, reinforced with graphene oxide (GOx) and cyclic arginylglycylaspartic acid (c-RGD). The objective was to create a multifunctional platform combining injectability, bioactivity, and structural stability. **Methods:** The OA/NSC/GOx-cRGD hydrogel was synthesized through Schiff base cross-linking (aldehyde-amine reaction). Characterization included FTIR (C=N bond at 1650 cm⁻¹), Raman spectroscopy (D/G bands at 1338/1567 cm⁻¹), SEM (porous microstructure), and rheological analysis (shear-thinning behavior). In vitro assays assessed fibroblast viability (MTT) and macrophage *TNF-α* secretion (ELISA), while ex-vivo injectability and retention were evaluated using chicken cardiac tissue. **Results:** The hydrogel exhibited shear-thinning behavior (viscosity: 10 to <1 Pa·s) and elastic-dominated mechanics (G′ > G″), ensuring injectability. SEM revealed an interconnected porous structure mimicking native extracellular matrix. Fibroblast viability remained ≥95%, and *TNF-α* secretion in macrophages decreased by 80% (30 vs. 150 pg/μL in controls), demonstrating biocompatibility and anti-inflammatory effects. The hydrogel adhered stably to cardiac tissue without leakage. **Conclusions:** The OA/NSC/GOx-cRGD composite integrates injectability, bioactivity, and structural stability, offering a promising scaffold for tissue regeneration. Its modular design allows further functionalization with peptides or growth factors. Future work will focus on translational applications, including scalability and optimization for dynamic biological environments.

## 1. Introduction

Biomaterials are designed to interact with biological systems to treat, improve, or replace human tissues, organs, or functions. These materials can be of natural origin, synthetic origin, or a combination of both, and are applied in various areas ranging from regenerative medicine to tissue engineering and medical devices. According to Ratner and coworkers, biomaterials must meet specific criteria, such as biocompatibility, biodegradability, and adequate mechanical properties, to ensure they do not provoke adverse responses in the organism. Biocompatibility refers to the ability of a material to integrate into the biological environment without inducing inflammatory or toxic reactions. Biodegradability, however, is crucial for certain biomaterials, especially those intended for temporary applications where controlled decomposition within the organism is anticipated [1]. Hydrogels have garnered significant interest in this area due to their three-dimensional structure, which allows for storing small molecules. Furthermore, their high biocompatibility is attributed to the high-water content present in their structural network, which reduces irritation caused by friction with the tissues in which they interact. Likewise, they reduce protein denaturation. Hydrogels are hydrophilic polymers that form a three-dimensional network capable of absorbing large amounts of water, allowing them to swell significantly without losing their shape until they reach their maximum degree of hydration or swelling index [2].

Tissue regeneration represents a critical frontier in modern medicine, addressing the limitations of traditional therapeutic approaches in treating complex injuries and degenerative conditions. The ability to effectively restore damaged tissues remains a significant challenge, particularly for complex biological systems that require intricate structural and functional reconstruction. Traditional approaches often fall short in providing optimal healing environments, necessitating the development of advanced biomaterials that can closely mimic the complexity of native tissue microenvironments. Injectable hydrogels have emerged as a revolutionary biomaterial class offering unprecedented potential in tissue regeneration strategies. These sophisticated materials combine the unique ability to be delivered minimally invasively with exceptional capacity to provide structural support, facilitate cellular interactions, and promote regenerative processes. Unlike traditional surgical interventions, injectable hydrogels can be precisely delivered through small needles, minimizing surgical trauma and enabling more precise therapeutic interventions.

While injectable hydrogels have various applications in biomedicine and other fields due to their unique properties mentioned above, in tissue regeneration, they can find use as three-dimensional matrices that favor the regeneration of damaged tissues due to their water retention capacity and because they promote a favorable environment for cell growth. Consequently, they are widely used to repair skin, bone, cartilage, and other tissues [3,4]. Alginate and chitosan, common polysaccharides already used for tissue engineering applications, are extensively employed in hydrogel formulations because they are biocompatible, chemically versatile, and structurally like components in the extracellular matrix [5].

Injectable hydrogels are suitable for tissue engineering applications due to their structural adaptability and capacity to mimic native extracellular matrix (ECM) environments [6]. Among natural polymers, alginate and chitosan are widely explored for their biocompatibility, biodegradability, and chemical versatility [7,8]. Alginate’s ability to form hydrogels via ionic cross-linking and chitosan’s inherent antibacterial properties make them ideal candidates for regenerative applications [9]. Recent advances include the use of chitosan/alginate hybrids for bone and dental tissue regeneration [10], wound healing [11], and spinal cord repair [12], although challenges persist in achieving a balance between mechanical strength, bioactivity, and injectability. Functionalization with bioactive peptides, such as cyclic peptide RGD (c-RGD), has shown promise in enhancing cell adhesion and tissue integration [13]. The demand for naturally occurring and engineered bioactive cyclic peptides is steadily rising [14,15,16,17,18], Different cyclic peptides containing an L-arginine-glycine-L-aspartic acid (cRGD) have been previously studied, enhancing cell adhesion compared to similar linear peptide sequences [19]. Interestingly, using hydrophobic moieties in the engineering design of the cyclic penta- and hexapeptide, including the RGD sequence, significantly improved the selectivity for the *αVβ3* integrin [20,21]. Similarly, graphene oxide (GOx) reinforcement improves mechanical properties and electrical conductivity, critical for cardiac and neural applications [22]. Recent studies highlight the efficacy of chitosan–alginate hydrogels enriched with ascorbic acid [23] or zinc oxide nanoparticles [24,25] for antioxidant and antibacterial activity, while self-cross-linkable oxidized alginate–carboxymethyl chitosan systems demonstrate potential for dental enamel regeneration [26]. However, few studies combine peptide functionalization, GOx reinforcement, and dynamic rheological optimization in a single platform [27,28].

Despite significant advancements in chitosan/alginate hydrogels for applications such as osteogenesis [29,30], intervertebral disk repair [31], and chronic wound healing [32], existing systems often prioritize single functional properties over multifunctional integration. On the other hand, 3D-printed chitosan/alginate/hydroxyapatite scaffolds [33] and SLN–insulin composites [34] address mechanical or permeability challenges but neglect anti-inflammatory and ECM-mimetic functionalities. This work fills a critical gap by unifying three pivotal attributes in a single platform: shear-thinning injectability via Schiff base cross-linking, mechanical reinforcement through GOx integration, and enhanced bioactivity via c-RGD peptide functionalization. Unlike prior studies that focus on isolated properties, our composite synergizes oxidized alginate’s degradability, *N*-succinyl chitosan’s solubility, and GOx’s conductivity with c-RGD’s cell-adhesive specificity, overcoming the trade-off between injectability and structural stability. The novelty lies in the covalent conjugation of c-RGD to GOx, ensuring sustained peptide presentation [35], and the use of a self-crosslinking OA/NSC network that eliminates toxic crosslinkers. This approach outperforms conventional ionic or UV-crosslinked hydrogels [10,26] by offering pH-responsive gelation, tunable porosity, and reduced immunogenicity, as evidenced by suppressed *TNF-α* secretion. By bridging material innovation with clinical needs, this work establishes next-generation multifunctional hydrogels in regenerative medicine.

## 2. Results and Discussion

### 2.1. Preparation and Characterization of Precursors and Hydrogel Composite

**Figure 1:**
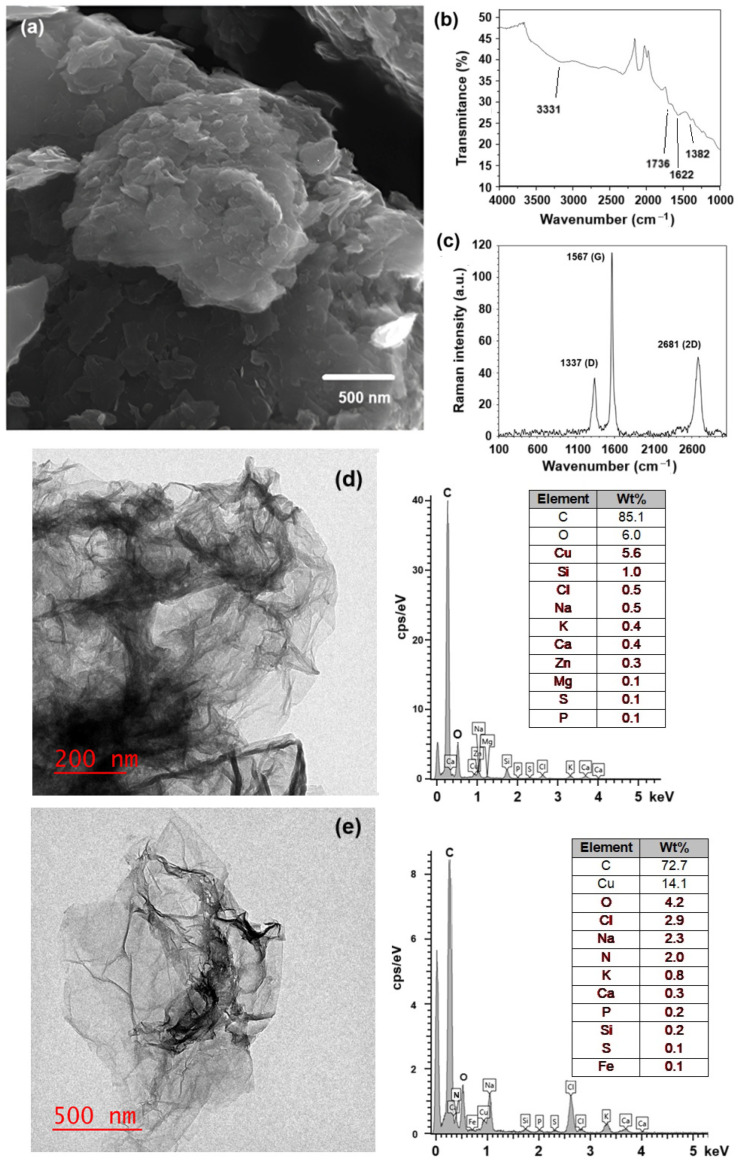
(**a**) SEM micrography of the prepared graphene oxide (Hummer’s method); (**b**) FTIR spectra indicating the band associated with carboxyl (*C*=*O*) groups; (**c**) Raman spectra confirming the presence of their characteristic D and G bands; (**d**,**e**) TEM micrographs of pristine GOx and c-RGD peptide conjugated to GOx, showing the corresponding chemical analysis obtained by EDX.

To prepare the hydrogel composite, first, each component (GOx, OA, NSC, and c-RGD) was prepared or chemically modified by following previously reported experimental procedures, as described in Section 3. The morphology of GOx, as obtained from the modified Hummer’s method, was determined by SEM and TEM. Figure 1a shows the characteristic aggregated layered sheets of GOx, with some individual sheets distinguishable. The prepared GOx flakes were randomly aggregated, forming a disordered solid. FTIR analysis of the synthesized GOx (Figure 1b) indicates the presence of a band at 3331 cm^−1^ corresponding to the tension vibrations of the hydroxyl group (*O–H*), another band at 1736 cm^−1^ typical of the deformation vibrations for the *C=O* group, and one band at 1622 cm^−1^ assigned to the deformation vibrations of the *C=C* group in the aromatic ring, with another band at 1382 cm^−1^ associated with the deformation vibrations of the epoxide groups (*C–O–C*); all these bands indicate the existence of an oxidized graphene-like structure [36,37,38,39]. The Raman spectra (Figure 1c) contain bands 1338 cm^−1^ (D band) and 1567 cm^−1^ (G band, E_2g_ mode), confirming the exfoliated, few-layer graphitic nature of the material. The shift from ~1600 cm^−1^ (typical for pristine graphite) to 1567 cm^−1^ confirms extensive oxidation of the graphite 2D network. The G band is associated with graphitic carbons, while the D band is related to structural defects or partially disordered graphitic domains [40]. TEM micrographs (Figure 1d,e) clearly show the laminar nature of the GOx and c-RGD peptides conjugated to GOx materials, respectively. EDS analysis reveals the presence of nitrogen in the GOx-peptide conjugated, while it was not present in pristine GOx, providing evidence of peptide functionalization.

**Figure 2:**
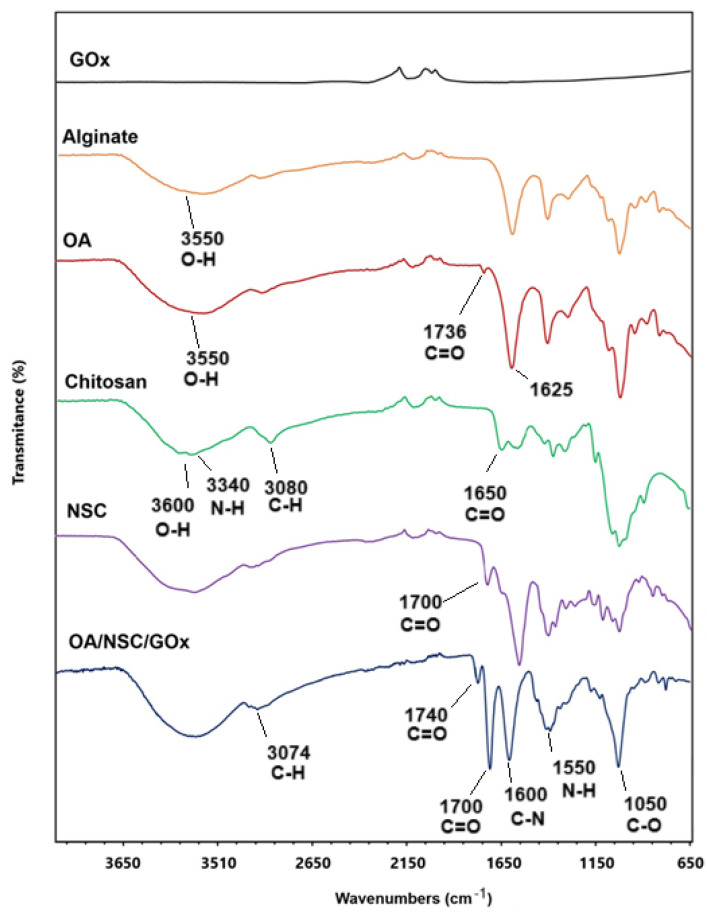
Comparative infrared spectrum of OA/NSC/GOx hydrogel composite and the individual pure components.

The synthetic pathway for preparing the oxidized alginate/*N*-succinyl chitosan/graphene oxide hydrogel composite (OA/NSC/GOx) is described in the experimental methodology. Oxidized alginate (OA) was prepared at room temperature. Confirmation of alginate oxidation was performed by FTIR, where a weak peak was present around 1736 cm^−1^, assigned to the symmetric vibration of the aldehyde group, which was absent in the vibrational spectra of pure sodium alginate (Figure 2). *N*-succinyl chitosan (NSC, a biocompatible derivative soluble in acidic aqueous media and obtained from the *N*-acylation of chitosan) was successfully prepared as previously described, and the FTIR spectra of NSC are shown in Figure 2, confirming the acetylation of the chitosan structure. NSC is an excellent component for developing hydrogel or nanoparticle-based formulations for drug delivery, as well as a versatile platform for peptide conjugation in tissue engineering scaffolds applications and wound dressings [41].

In the preparation of OA, the resulting material is expected to present certain distinctive spectral characteristics in the infrared spectrum. When oxidized, the alginate structure tends to generate more hydroxyl groups (*–OH*) and carbonyl (*C=O*) groups, as new functional groups are formed when bonds are broken in the polysaccharide skeleton. This was confirmed by the hydroxyl group (*–OH*) stretching bands appearing in the 3200 to 3550 cm^−1^ region and the *C=O* stretch band present at 1625 cm^−1^ [42,43]. The IR spectrum revealed notable differences between chitosan and NSC. In chitosan, we can observe a broad band around 3400 cm^−1^ due to *O–H* stretching and hydrogen bonding, a band around 3300–3200 cm^−1^ characteristic of *N–H* stretching, and a weak signal at 1650 cm^−1^ corresponding to the amide. In the chemically modified product, the hydroxyl band was slightly altered, possibly due to the interaction of the new carboxyl groups with the hydroxyl groups. The *N–H* band weakened or overlapped with others due to the modification in the amino group; a new, intense band appeared near 1700 cm^−1^, attributed to the *C=O* stretching, indicating the successful modification. Finally, both substances presented *C–H* stretching at 2900–2800 cm^−1^ and *C–O–C* vibrations at 1000–1100 cm^−1^ [44,45]. For the OA/NSC/GOx hydrogel composite, the IR spectrum showed a broad band at 3300–3400 cm^−1^ associated with the stretching vibration of the *-OH* groups present in the hydrogel formulation. The interaction of the *-OH* groups in GOx and alginate could contribute to the mechanical and water retention properties of the material. The band in the region of 2900 cm^−1^ represents the stretching vibrations of the *–CH_2_* groups (methylene groups), common in the structure of polysaccharides such as alginate and chitosan. This signal is characteristic of hydrocarbon skeletons and confirms the presence of the base components in the hydrogel. The band at 1720 cm^−1^ corresponded to the *C=O* stretching vibration of the carboxylic groups in GOx and OA, while the band in the 1650 cm^−1^ region could be associated with the Schiff (*C=N*) bonds formed between the aldehyde group of the OA and the amino group of NSC. The appearance of this band is fundamental, as it confirms the formation of Schiff cross-links, which are responsible for the cross-linked structure of the hydrogel, providing mechanical stability and controlled degradation. This is further supported by the characteristic band of the *N–H* bending vibrations of amides between 1550 and 1600 cm^−1^. The band at 1400 cm^−1^ can be associated with the vibration of the *COO^−^* groups in the alginate, indicating the presence of carboxylate groups. Finally, the band at 1100–1150 cm^−1^ is related to the *C–O–C* and *C–OH* stretching vibrations, both in the GOx and the glycosidic bonds of the alginate and chitosan [46,47,48,49,50,51,52,53,54,55,56].

**Figure 3:**
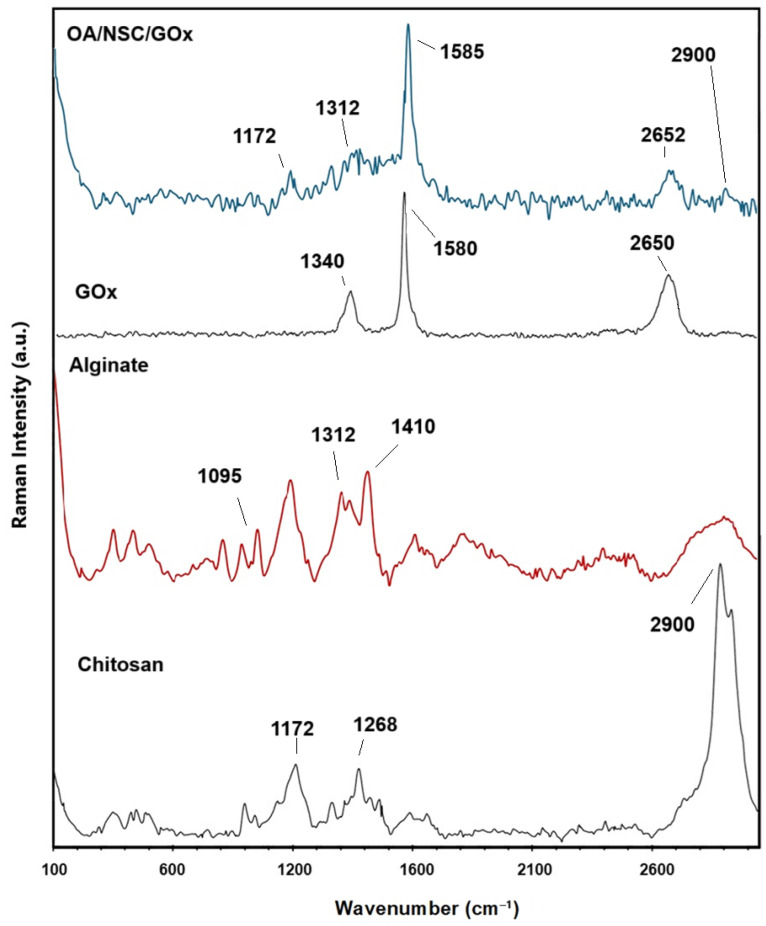
Comparative Raman spectra for chitosan, alginate, graphene oxide (GOx), and the OA/NSC/GOx hydrogel composite.

Raman spectroscopy analyses were performed to prove the formation of the cross-linked OA/NSC/GOx hydrogel composite. Figure 3 shows a comparison of the Raman spectra for chitosan, sodium alginate, GOx, and the OA/NSC/GOx hydrogel composite. The predominant band present in the chitosan Raman spectra was a strong peak at 2900 cm^−1^; from *–CH_2_* groups stretching, 1172 cm^−1^ was assigned to *CH_3_-* wagging, and 1268 cm^−1^ was assigned to *CH_2_* twisting. For alginate, the Raman spectrum showed bands around 1410 cm^−1^ (carboxylate stretching vibration), 1312 cm^−1^ (*C–O* stretching vibration), and 1095 cm^−1^ (glycosidic ring breathing mode). The D band at 1330–1340 cm^−1^ and G band near 1580–1600 cm^−1^, referring to the A_1g_ and E_2g_ modes of sp^2^ carbon atoms, were present either at pristine GOx or the OA/NSC/GOx hydrogel composite, confirming the successful incorporation of GOx into the hydrogel composite network.

**Figure 4:**
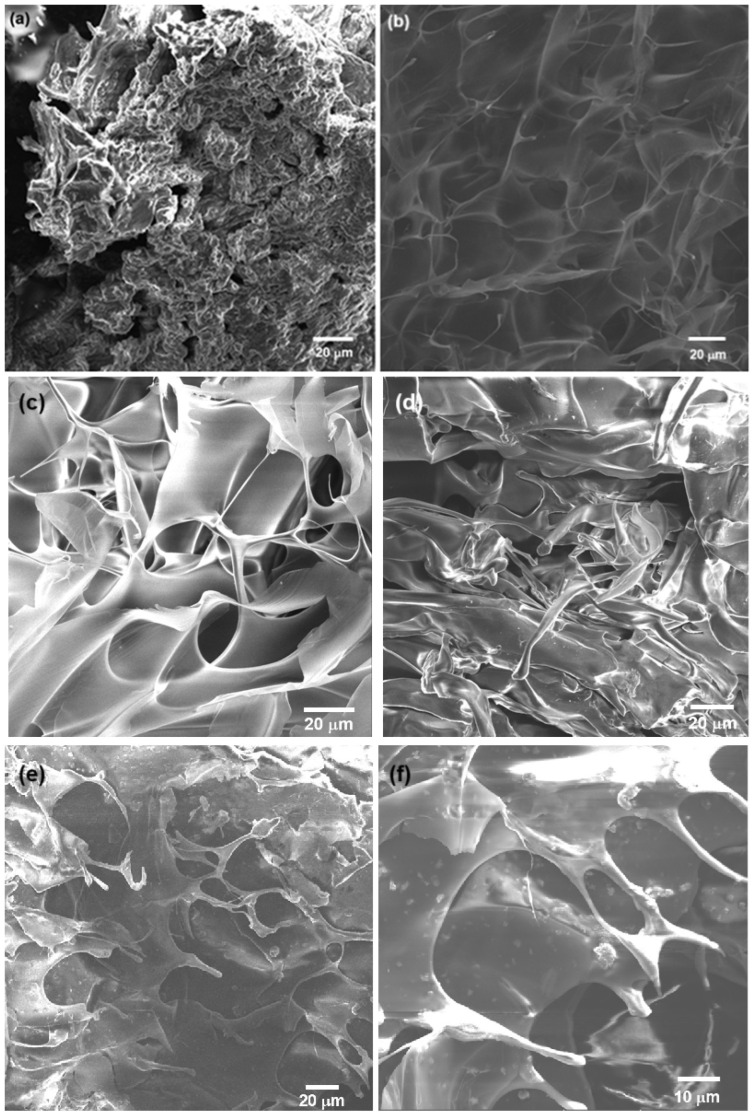
SEM images of 3% hydrogel of (**a**) chitosan and (**b**) *N*-succinyl-chitosan; (**c**) 3% alginate hydrogel and (**d**) oxidized alginate (OA); (**e**) crosslinked OA/NSC hydrogel and (**f**) OA/NSC/GOx hydrogel composite. An interconnected, porous structure with small pore size was observed. GOx flakes were visible on the surface of the OA/NSC/GOx hydrogel composite.

The SEM analysis revealed that the 3% chitosan hydrogel presented a 3D, porous, rough structure (Figure 4a). The macropore surfaces were irregular, and the material appeared to have a disordered and compact arrangement. These characteristics were typical of chitosan hydrogels, which typically have a porous matrix when dried [52]. For the modified NSC hydrogel (Figure 4b), the SEM analysis of the freeze-dried, lyophilized sample showed a continuous, 3D porous structure, with thin and wavy sheets, with larger pore size compared to unmodified chitosan. The hydrogel retained its porous network structure after the functionalization. NSC has characteristics that make it more flexible and with greater absorption capacity, which is useful in biomedical applications, since it can improve biocompatibility and cellular response. For example, the presence of amino and hydroxyl groups facilitates the interaction of the NSC hydrogels with metal ions, forming non-covalent and covalent interactions and promoting the formation of three-dimensional crosslinked mesh structures; also, improved water retention makes the hydrogels soft and flexible. Increasing the content of NSC into a hydrogel composite enhances swelling and gives the hydrogel pH-sensitive properties [54,55,56,57].

Figure 4c shows the morphology of the pristine 3% alginate hydrogel, where an organized, rough structure was present, with a well-defined pore network. Large pores and thin walls are typical in pure alginate hydrogels, which have a more uniform and symmetrical structure [58]. For the oxidized alginate hydrogel (Figure 4d), a structure was observed that appeared less ordered and more intertwined, with irregular fibers and fragments. Alginate oxidation introduced changes in structure, such as greater degradability and lower mechanical strength, but also increased the ability to form cross-links with other polymers or biological agents, expanding its use in applications where there is greater biocompatibility and reabsorption in the body [58]. Finally, the combination of the OA and the NSC biopolymers was achieved by Schiff base condensation, resulting in a cross-linked hydrogel composite. After lyophilization, the sample was analyzed by SEM (Figure 4e). The obtained hydrogel displayed a continuous porous network structure, showing a mixed microstructure with morphological similarities to that of the individual components (Figure 4a,c). The cross-linked OA/NSC hydrogel had a rough and wrinkled surface. The addition of 0.2% of GOx to the cross-linked hydrogel resulted in the formation of an OA/NSC/GOx hydrogel composite (Figure 4f). Raman spectroscopy confirmed that the GOx was present and dispersed in the composite’s structure (Figure 3). It showed the same wrinkled and porous morphology as the cross-linked OA/NSC hydrogel, with no visible structural changes due to the presence of the GOx platelets.

**Figure 5:**
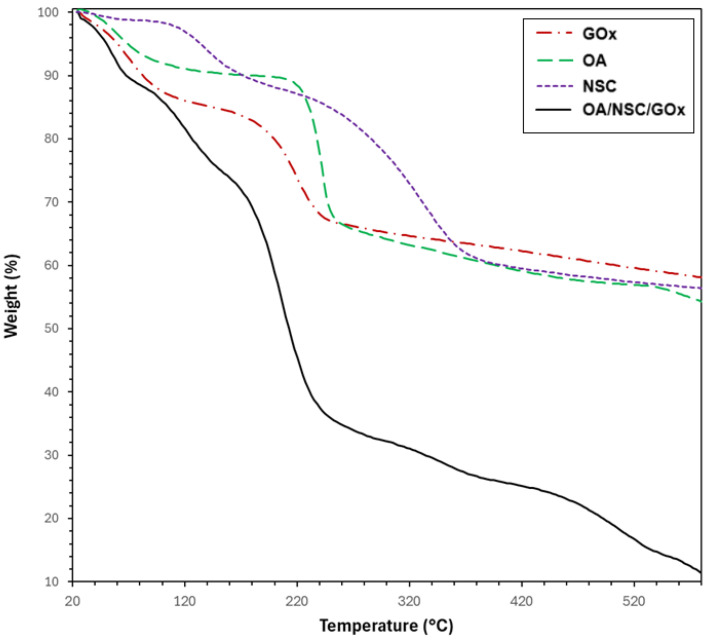
TGA thermograms for GOx, alginate, chitosan, and the hydrogel composite OA-NSC/GOx.

Figure 5 displays the TGA curves of the hydrogel composite (OA/NSC/GOx) compared with its components (GOx, OA, and NSC). The TGA curves corresponding to unmodified alginate and chitosan are available in the Supplementary Information Material. The TGA curve for GOx (discontinuous red line) exhibited two thermal processes: the first occurring up to 100 °C (weight loss of approximately 15%), attributable to water evaporation, followed by a second weight loss (~17%) from 100 to 250 °C, likely related to the thermal decomposition of carboxylic (*–COOH*) and hydroxyl (*–OH*) groups. The TGA curve for pure chitosan (see Supplementary Information) also presented two thermal stages of weight loss: the first occurred in the 47–100 °C range (water evaporation, ~9% weight loss), followed by primary thermal degradation of chitosan beginning around 240 °C, with major decomposition occurring near 296 °C and continuing beyond 450 °C, resulting in a weight loss of approximately 53%. In contrast, NSC presented three thermal processes: the first one corresponding to water evaporation (~2% weight loss, <120 °C), followed by a second process in the 120–220 °C temperature range (12% weight loss) and attributed to the degradation of the polysaccharide backbone; a third process occurring from 220 to 430 °C (34% weight loss) was associated with the thermal degradation of the succinyl group [59]. It was calculated that the *N*-succinylation degree, from the TGA data, was around 34%. For unmodified alginate, the TGA curve (see Supplementary Information) revealed two consecutive thermal steps. The first weight loss, up to 100 °C (~18%), was likely due to water removal, and a second loss (~45%), between 220 °C and over 450 °C, resulted from the complex thermal decomposition of saccharide rings and macromolecule chains. OA presented a similar TGA curve, showing also two consecutive thermal processes; the first one with water evaporation under 100° (4% weight loss), followed by a sharp weight loss (~26%, 210–280 °C) attributable to the thermal degradation of the polysaccharide chain, followed by a steady weight loss process until 600 °C (weight loss ~8%). However, the thermal process occurring in the 210–280 °C range was slightly shifted to a higher temperature range, probably due to an induced thermal protection effect associated with the formation of stable inter-residue hemiacetals between an aldehyde and a hydroxyl group after oxidation of the alginate chain [59]. The estimated oxidation degree for OA was calculated to be ~45%. Finally, the OA/NSC/GOx hydrogel composite showed a weight loss (~11%) before 90 °C, resulting from the evaporation of water molecules trapped in the hydrogel’s porous structure, followed by a second weight loss (~22%) between 90 and 170 °C due decarboxylation and thermal degradation of OA. A third stage with a weight loss of 38% in the 170–320 °C range can be attributed to the decomposition of cross-linking of the hydrogel composite and deacetylation of the NSC component. Similar thermal behavior for an NSC derivative was previously reported [60]. The gradual weight loss processes after 320 °C may be attributed to the continued thermal decomposition of the hydrogel composite.

### 2.2. Rheology and Mechanical Properties of the Composite Hydrogel

**Figure 6:**
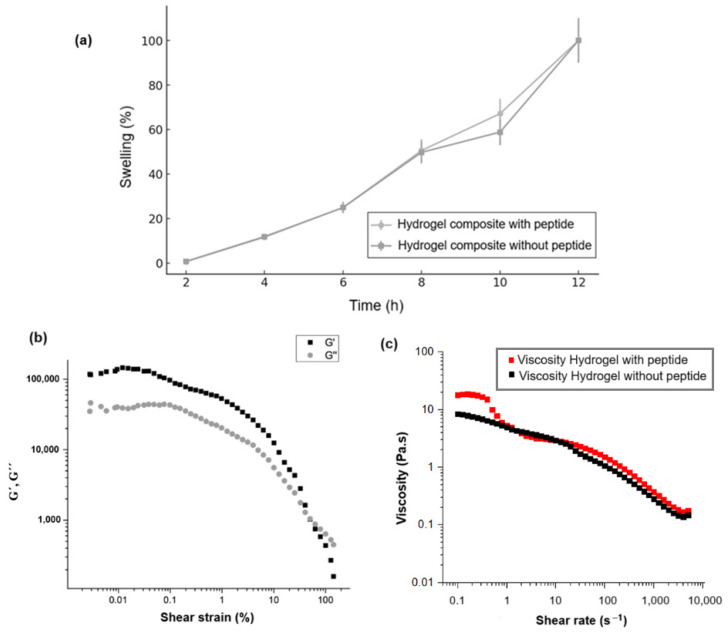
(**a**) Swelling behavior for the hydrogel composite with and without peptide immobilization. (**b**) Variations of storage modulus (G′) and loss modulus (G″) with shear stress of the OA/NSC/cRGD-GOx hydrogel composite. (**c**) Shear rate–viscosity curve of the hydrogel composite with and without the peptide.

Figure 6 illustrates the rheological properties of the OA/NSC/GOx hydrogel composite, before and after peptide modification. Swelling reflects changes in the volume of a hydrogel when it absorbs water. Figure 6a compares the swelling behavior for the hydrogel composite with and without peptide immobilization. In both cases, the hydrogels exhibited a linear increase in swelling with respect to time, not presenting significant differences among them. Figure 6b shows specifically the variation of storage modulus (G′) and loss modulus (G″). As established in the literature, G′ (storage modulus) represents the material’s capacity to store elastic energy when deformed, corresponding to the solid or elastic behavior of the hydrogel, while G″ (loss modulus) indicates the material’s ability to dissipate energy through viscous flow, reflecting the liquid or viscous behavior of the hydrogel [61,62,63]. The data demonstrate that G′ > G″ throughout most of the deformation range, indicating that the hydrogel exhibited predominantly elastic behavior, functioning more like a solid than a liquid under small deformations. However, both moduli decreased as strain increased, beginning at 10% strain, which characterized “shear-thinning”. This behavior is typical in hydrogels that experience a breakdown of their internal structure under large deformations, resulting in decreased rigidity and increased fluidity. Therefore, the hydrogel displayed dominant elastic behavior at low strains. As the strain increased, its structure progressively weakened, suggesting hydrogel may be susceptible to rupture or degradation of its internal network under high-stress conditions [64,65,66]. As Figure 6b demonstrates, the hydrogel maintained a predominantly elastic phase (G′ > G″) across a wide range of small deformations, indicating sufficient stiffness to provide structural support to soft tissues, a beneficial characteristic for biomedical applications. The material also exhibited weakening behavior under high strains (significant reduction in G′ and G″), which is advantageous for applications requiring adaptability and deformation capabilities under stress, such as during injection or when filling irregular spaces. This suggests the hydrogel is sufficiently stable to maintain its shape while remaining flexible enough to adapt to the dynamics of soft tissues [67,68,69,70]. Figure 6c illustrates the relationship between viscosity (Pa·s) and shear rate (s^−1^). The viscosity decreased from approximately 10 Pa·s at lower shear rates to below 1 Pa·s as the shear rate increased, across a broad range from approximately 0.1 to 10,000 s^−1^. This curve demonstrated that the hydrogel exhibited shear-thinning behavior, a beneficial characteristic for injectable applications as it reduces viscosity under shear while maintaining structural integrity afterward. The addition of the peptide did not seem to have an impact on the viscosity of the hydrogels. For injectability, a viscosity of 10 Pa·s was generally favorable, allowing the hydrogel to flow smoothly through a needle or catheter. This shear-thinning property is particularly advantageous during injection, enabling administration with reduced force [71]. However, while lower viscosity facilitates easier injections, it may also predispose the hydrogel to spreading or leaking from the injection site, particularly in highly dynamic environments such as cardiac tissue [72]. A viscosity of 10 Pa·s may be considered acceptable for injectable hydrogels in applications where retention and mechanical support are less critical factors [73]. In summary, peptide immobilization on the GOx used to reinforce the hydrogel composite did not affect its rheological and mechanical properties.

### 2.3. Qualitative Injectability Process

**Figure 7:**
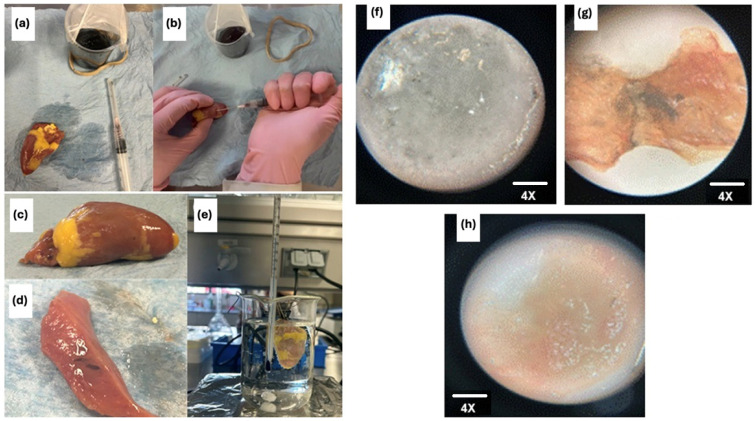
Samples under the microscope at 4×. (**a**) shows the hydrogel composite; (**b**) shows the chicken cardiac tissue after the hydrogel composite injection; (**c**) shows the chicken cardiac tissue before hydrogel composite application; (**d**) shows the hydrogel composite in a longitudinal section of the tissue; (**e**) shows the experimental setup with the heart in a physiological solution with constant stirring; (**f**) hydrogel composite 20×; (**g**) hydrogel composite on a chicken heart tissue slice; (**h**) chicken heart tissue slice.

Injectable hydrogels hold unique promise for tissue regeneration applications by serving as artificial three-dimensional cellular matrices that function as bioactive scaffolds for cell anchorage. Natural polymers such as alginate, hyaluronic acid, and chitosan—whose chemical structures mimic native glycosaminoglycans (GAGs)—have been widely explored for hydrogel fabrication due to their biocompatibility and bioactivity [74]. These polymers feature abundant functional groups that facilitate cell adhesion and modulate cellular behavior, though achieving precise control over their mechanical properties remains a technical challenge [75]. The hydrogel composite developed in this study exhibits superior rheological performance, significantly enhancing its injectability and suitability for tissue regeneration. Prior studies highlight the therapeutic potential of alginate–chitosan hydrogels in regenerative medicine: their injection has been shown to promote bone and cartilage repair, stimulate myocardial regeneration, reduce scar tissue formation, enhance angiogenesis, and potentially activate endogenous cardiomyocyte proliferation while recruiting cardiac stem cells [76,77]. These findings underscore the versatility of such hydrogels in addressing complex tissue repair processes. As the prepared hydrogel composite exhibits superior rheological properties (appropriate stiffness for supporting soft tissues, flexibility to adapt and deform under flow or stress, and low viscosity), it demonstrates suitable injectability characteristics. A preliminary qualitative test was conducted to evaluate the injectability of the prepared OA/NSC/GOx hydrogel composite. A 0.2 mL volume of the hydrogel (Figure 7a–e) was injected into a section of a chicken heart tissue, which was subsequently placed in a physiological solution with constant agitation for 2 h to detect any potential leakage of the hydrogel composite into the medium. Under microscopic examination (4×), the hydrogel composite (Figure 7f) presents a clear appearance with amorphous morphology. The chicken heart tissue was then sectioned in the injection site to reveal the persistence of the hydrogel composite within the tissue (Figure 7g). Good adhesion of the hydrogel composite within the chicken heart tissue was observed. For comparison, normal chicken heart tissue, without application of the hydrogel composite, was also examined under the microscope (4×), showing no color or morphological alterations (Figure 7h). The hydrogel composite exhibited a complex, porous microstructure (Figure 7d) that resembles the 3D structural network of a biological tissue.

### 2.4. In Vitro Essays

**Figure 8:**
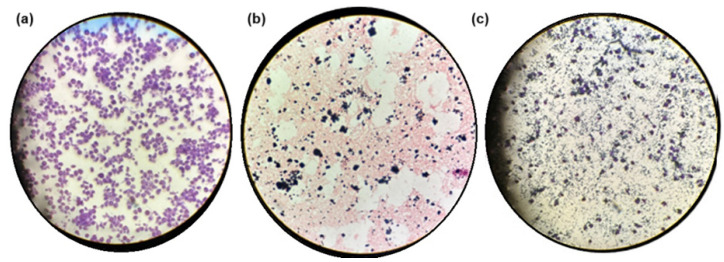
Samples under the microscope at 10×. (**a**) Macrophages in culture media; (**b**) hydrogel composite in culture media; (**c**) combination of macrophages and hydrogel composite in the same culture media.

We examined the interaction between macrophage cell cultures and the hydrogel composite. Figure 8a and 8b show macrophages and the hydrogel composite dispersed separately in pure culture media, respectively. Purple-colored, round to oval-shaped cells with diameters ranging from 10 to 30 mm (macrophages) were visible (Figure 8a). Black aggregates (GOx) were randomly distributed throughout the complex 3D porous structure of the pink-colored hydrogel composite (Figure 8b). Figure 8c shows the integrated system containing macrophages (large purple oval structures), GOx black aggregates, and the hydrogel composite (light purple disordered structures) combined in the culture media. Macrophages were distributed primarily within the porous structure of the hydrogel composite, alongside the GOx aggregates. The biocompatible, porous hydrogel composite, with its 3D structure that mimics the extracellular matrix, appeared to provide an appropriate environment for hosting cells within its void spaces and offered sites for cellular adhesion, facilitating nutrient and oxygen diffusion, as well as supporting cell proliferation.

**Figure 9:**
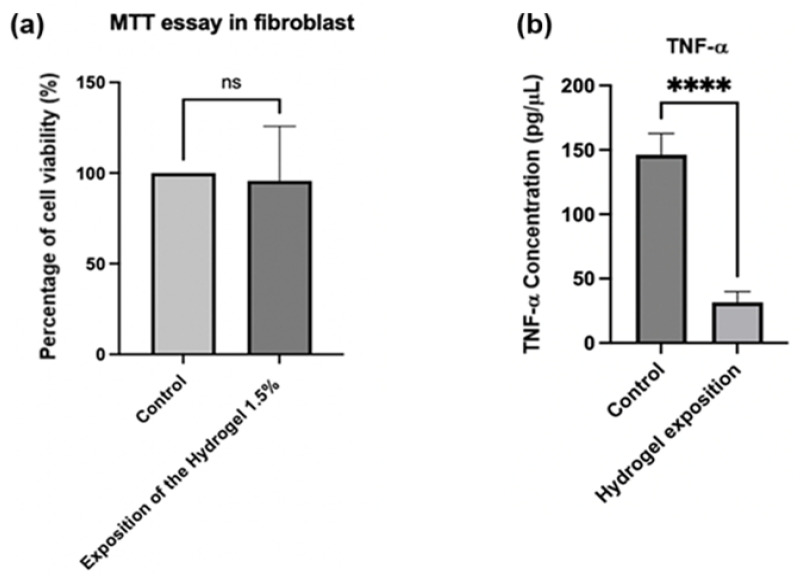
(**a**) Percentage of cell viability for fibroblasts exposed to hydrogel. *p* values were calculated using an unpaired *t*-test for a single variance. (**b**) Concentration of TNF-α for the exposition of fibroblasts into the hydrogel. *p*-values were calculated via *t*-test for unpaired data for a single variance. **** *p* = 0.0001, ns: not significant.

Finally, Figure 9a presents the results of a cell viability test (MTT) with fibroblasts, evaluating the effect of a 1.5% injectable hydrogel composite compared to a control group. The group treated with the 1.5% hydrogel composite demonstrated no significant decrease in cell viability relative to the control group. This suggests that exposure to hydrogel at this concentration does not exert cytotoxic effects on fibroblasts [78,79,80]. TNF-α is an established inflammatory cytokine [81], and its reduction may indicate an anti-inflammatory effect of the hydrogel. Figure 9b demonstrates that when implementing the treatment described above to obtain macrophages through PMA-induced differentiation, the “control” group (macrophages without hydrogel exposure) exhibited TNF-α concentrations of approximately 150 pg/μL. In contrast, the group exposed to the hydrogel showed significantly lower concentrations, approximately 30 pg/μL. Therefore, exposure of the differentiated macrophages to hydrogel substantially reduced TNF-α levels compared to the control group. This analysis was conducted using an ELISA kit [82,83].

The OA/NSC/GOx-cRGD composite hydrogel developed in this work overcomes the limitations of conventional injectable systems by integrating rheological, mechanical, and bioactive properties into a single platform. Unlike thermo-responsive gelatin–methacrylate hydrogels, which require UV light for crosslinking [84], our system employs self-healing Schiff base bonds, eliminating the need for external stimuli and reducing cytotoxicity. Furthermore, the shear-thinning behavior (viscosity from 10 to <1 Pa s) allows for good injection, comparable to that of peptide-modified polyethylene glycol (PEG) hydrogels [85], but with the added advantage of a porous microstructure (50–200 μm) that facilitates cell migration, unlike dense networks such as those of covalently crosslinked hyaluronic acid hydrogels [86]. The integration of c-RGD-functionalized GOx sustainably improves cell adhesion, outperforming systems such as silver-doped alginate hydrogels, which prioritize antibacterial properties but lack cell-targeting [87]. The 80% reduction in TNF-α observed in macrophages suggests superior anti-inflammatory activity compared to dexamethasone-loaded chitosan/hyaluronic acid hydrogels [88], highlighting the potential of GOx-cRGD to modulate inflammatory microenvironments in damaged tissues.

However, unlike injectable hydrogels tested in rodent myocardial infarction models [89], this study is limited to ex vivo testing in avian cardiac tissue, underscoring the need to assess long-term stability in vivo. Future studies should explore functionalization with angiogenic peptides (e.g., SDF-1α) to promote vascularization, inspired by fibrin/polylactic acid hydrogels used in bone tissue regeneration [90], underscoring the need to validate degradation and regenerative efficacy in complex physiological environments. Future studies should explore functionalization with proangiogenic peptides inspired by synergistic chitosan–alginate hydrogels enriched with ascorbic acid [23] to address vascularization in ischemic tissues.

## 3. Materials and Methods

### 3.1. Materials

Graphite, sodium nitrate (NaNO_3_), sulfuric acid (H_2_SO_4_, 98%, 5), potassium permanganate (KMnO_4_), distilled water, hydrogen peroxide (H_2_O_2_), and hydrochloric acid (HCl, 10%) were used. All reagents and solvents were reactive grade and were purchased from Merck-Sigma Aldrich (St. Louis, MO, USA): medium molecular weight chitosan (desacetylated chitin from shrimp shells, harvested in Iceland, deacetylation degree of 75–85%, minimum purity of 75%, Lot # STBF3507V, CAS 9012-76-4, cat. 448877, molecular weight ~190,000–310,000 Da), graphite (CAS 7782-42-5, cat. 496588, molecular weight 12.01 g/mol), medium viscosity sodium alginate (from brown algae, mannuronic acid to guluronic acid ratio ~1.56, Lot # SLCJ8300, CAS 9005-38-3, cat. A2033, repeating unit 198.11 g/mol, molecular weight 100,000–200,000 Da), lactic acid (Batch # SLCN1893, CAS 50-21-5, cat. 69785, 90.08 g/mol), succinic anhydride (CAS 108-30-5, cat. 239690, 100.07 g/mol), sodium chloride (CAS 7647-14-5, cat. S9888, 58.44 g/mol), ethylene glycol (CAS 107-21-1, cat. 324558, 62.07 g/mol), and sodium periodate (CAS 7790-28-5, cat. 311448, 213.89 g/mol). All chemicals were used without further purification. Fmoc-Arg(Pbf)-OH, Fmoc-Ahx-OH, Fmoc-Gly-OH, Fmoc-Asp(Ompe)-OH, Fmoc-Lys(Alloc)-OH, and Fmoc-Glu(OAll)-OH amino acids, as well as Fmoc-Lys(Boc)-Wang resin and Oxyma Pure, were purchased from CEM and used as received. Tetrakis(triphenylphosphine)palladium(0) [Pd(PPh_3_)_4_] was purchased from AK Scientific. *N*,*N*′-Diisopropylcarbodiimide (DIC), trifluoroacetic acid (TFA, HPLC grade), formic acid (HPLC grade), dimethylformamide (DMF, HPLC grade), piperidine (HPLC grade), acetonitrile (HPLC grade), triisopropylsilane (TIS), and phenylsilane (97%) were purchased from Sigma-Aldrich (St. Louis, MO, USA). All reagents and solvents were used as received.

### 3.2. Preparation of Graphene Oxide (GOx)

**Scheme 1:**
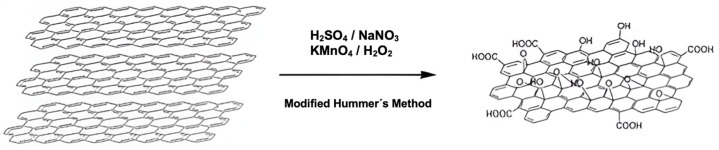
Modified Hummer’s method for the preparation of graphene oxide (GOx).

For the synthesis, the method described by Yu et al. was followed, with some modifications (Scheme 1) [91]. Initially, 2 g of graphite and 2 g of NaNO_3_ were mixed in 50 mL of H_2_SO_4_ (98%). The mixture was kept in an ice bath at a controlled temperature of 0–5 °C, with constant stirring. Subsequently, 6 g of KMnO_4_ was slowly added to the suspension, ensuring that the temperature did not exceed 15 °C. After completing the addition of KMnO_4_, the mixture was removed from the ice bath and maintained with stirring at 35 °C. The reaction continued until a pasty brown mixture was obtained, which was stirred for 2 days. Subsequently, the mixture was diluted by slowly adding 100 mL of distilled water, resulting in a color change accompanied by effervescence and a rapid increase in temperature (approximately 98 °C). An additional 200 mL of distilled water was added to the mixture under continuous stirring. Finally, the solution was treated with 10 mL of H_2_O_2_, which caused the solution color to change to yellow. The obtained solution was subjected to a washing process through three successive washes with HCl (10%) and, subsequently, with deionized water. Finally, the product was filtered and dried under vacuum at room temperature.

### 3.3. Synthesis of Oxidized Alginate (OA)

**Scheme 2:**
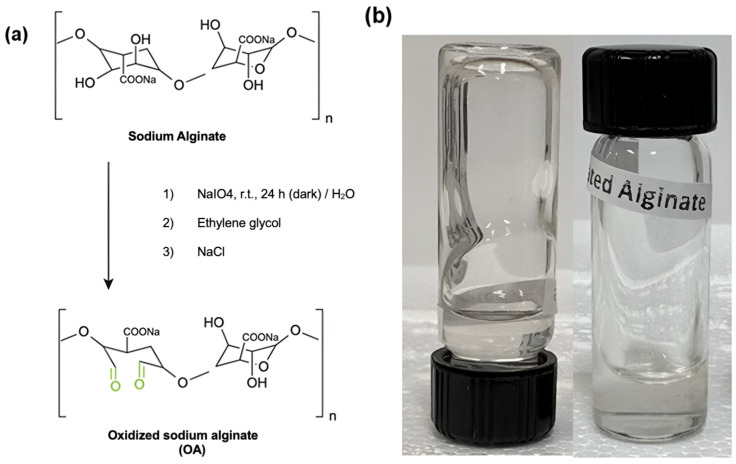
(**a**) Oxidized sodium alginate (OA) synthesis. (**b**) Picture of the OA hydrogel.

Oxidized alginate was synthesized following the previously reported procedure (Scheme 2) [92]. A total of 3.0 g of sodium alginate was dissolved in 150 mL of ultrapure water under constant stirring, obtaining a 2% (*w*/*v*) solution. Subsequently, an aqueous solution of sodium periodate (3.0 g in 10 mL) was slowly added to the alginate solution, stirring for 24 h at room temperature and in the dark. A total of 2 mL of ethylene glycol was added to the mixture to stop the oxidation reaction. Then, 1 g of sodium chloride was dissolved in the reaction product, and the solution was purified using dialysis membranes (MWCO 14,000) in ultrapure water for 3 days. Finally, the purified solution was frozen at −83 °C and lyophilized in a FreeZone 2.5 Plus benchtop freeze dryer (Labconco, Kansas City, MO, USA) at −40 °C and 0.22 mBar. According to the reported experimental procedure followed for the preparation of OA, the oxidation degree of sodium alginate prepared by this method was ~44.5%.

### 3.4. Synthesis of N-Succinyl Chitosan (NSC)

**Scheme 3:**
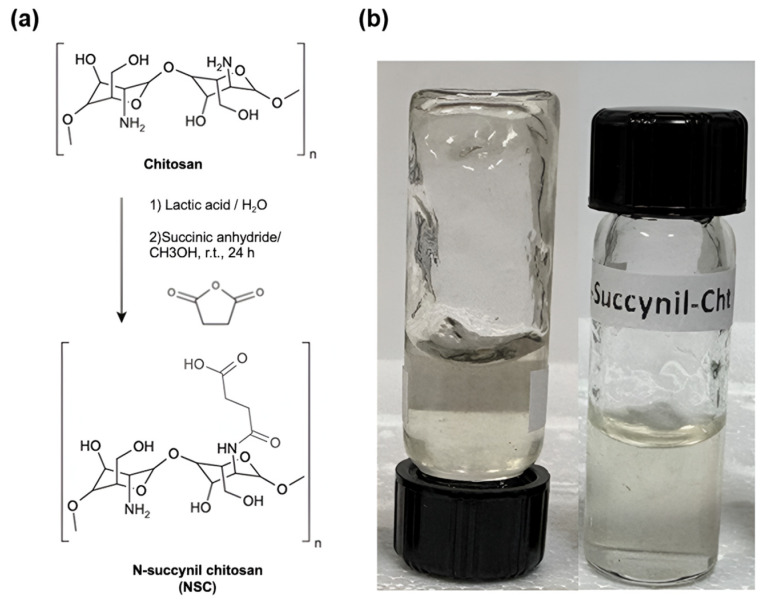
(**a**) *N*-succinyl chitosan (NSC) synthesis. (**b**) Picture of the NSC hydrogel.

The synthesis of *N*-succinyl chitosan was carried out following a previously reported procedure, with slight modifications (Scheme 3) [93]. A total of 0.5 g of medium molecular weight chitosan was dissolved in 28.5 mL of deionized water, adding 1.5 mL of lactic acid. The mixture was stirred for 2 h until the chitosan was completely dissolved. Subsequently, 120 mL of methyl alcohol and 1.5 g of succinic anhydride were added to the solution. This mixture was stirred at room temperature for 24 h. The pH of the solution was then adjusted to approximately 7 using a 5% (*w*/*v*) NaOH solution. The precipitated product was purified by dialysis with 14,000 MWCO for 3 days. Finally, the product obtained was lyophilized as previously described. This experimental procedure reported a *N*-succinylation degree on chitosan of 36.8%.

### 3.5. Synthesis of c-RGD-Ahx-K Peptide

**Scheme 4:**
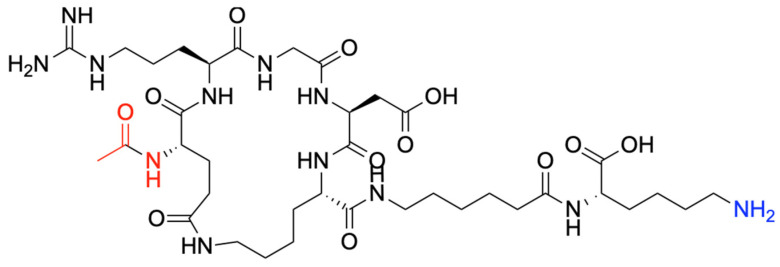
Molecular structure of the c-RGD-Ahx-K peptide.

In a Liberty Blue automated system, the cyclic de novo-designed peptide was synthesized using microwave-assisted solid-phase peptide synthesis (SPPS) (Scheme 4) [94]. Briefly, Wang Lys-resin was swelled in DMF for 5 min. Next, Fmoc deprotection was performed with 20% piperidine at 60 °C for 180 s. To add Ahx, Glu, Gly, and Arg to the peptide sequence, standard *N*,*N*′-DIC/Oxyma Pure coupling cycles were run at 60 °C for 360 s. The Arg motif was added using double coupling cycles, twice at 60 °C for 360 s. The special amino acids Lys(Alloc) and Glu(OAllyl) were simultaneously deprotected, and further peptide stapling via lactamization occurred as follows [95,96]. First, the simultaneous deprotection took place using 0.025 M Pd(PPh_3_)_4_ and phenylsilane 0.5 M in DCM under an argon atmosphere. Then, the side-chain stapling was conducted using 0.34 M DIC and 0.17 M HOBt in DMF under microwave at 90 °C for 600 s. The product was cleaved from the resin and deprotected with TFA/TIS/H_2_O (95/2.5/2.5% *v*/*v*/*v*) at 42 °C for 60 min and then precipitated using −20 °C diethyl ether. The crude peptide was then dried under nitrogen overnight and redispersed in Phase A. Then, the crude was purified by High-Performance Liquid Chromatography (HPLC) in an Agilent 1290 Infinity II Preparative LC/MSD System in Preparative Mode, with a 100 × 30 mm C18 column at 50 mL/min. A gradient from 0% to 40% phase B over 12 min was applied at a 50 mL/min flow rate. Phase A consisted of water/acetonitrile (5%/95%) with 0.1% formic acid and 0.01% TFA, and phase B was acetonitrile/water (98%/2%) with 0.1% formic acid and 0.01% TFA. A purity of >95% was determined through HPLC peak analysis. Finally, the c-RGD-Ahx-K peptide (Scheme 4) was freeze-dried for 72 h and stored for further characterization.

### 3.6. c-RGD Grafted Graphene Oxide (c-RGD-GOx)

**Scheme 5:**
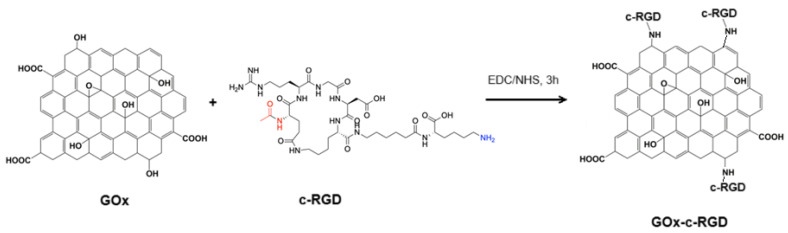
Conjugation of c-RGD on GOx by the EDC/NHS coupling protocol.

Covalent immobilization of the c-RGD-Ahx-K peptide to graphene oxide was achieved by following the well-known NHS/EDC coupling amidation reaction to form RGD-GOx nanomaterial (Scheme 5) [35]. In summary, 100 mL of GOx aqueous suspension (2.0 mg/mL) was mixed with 100 mL of an aqueous solution containing 20 mmol (3.84 mg) EDC and 20 mmol (2.30 mg) NHS and left to react for 3 h. After that, 2 mmol (1.74 mg) of the c-RGD peptide solution was added and incubated for 4 h. After this time, the black suspension was centrifuged (3000 rpm) at 4 °C, the over natant was discarded, and the solid pellet was dried under vacuum at 60 °C and refrigerated until it was further required.

### 3.7. Preparation of the OA-NSC/GOx or OA-NSC/cRGD-GOx Hydrogel Composite

**Scheme 6:**
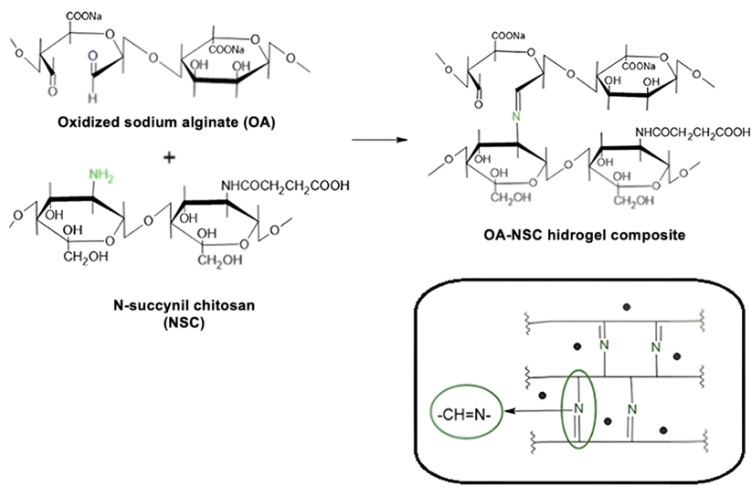
Schematic illustration of the cross-linking process between oxidized sodium alginate and *N*-succinyl-chitosan (Schiff base condensation). This reaction gives rise to a cross-linked three-dimensional structure, where the alginate and chitosan molecules are linked through Schiff base-type covalent bonds.

**Scheme 7:**
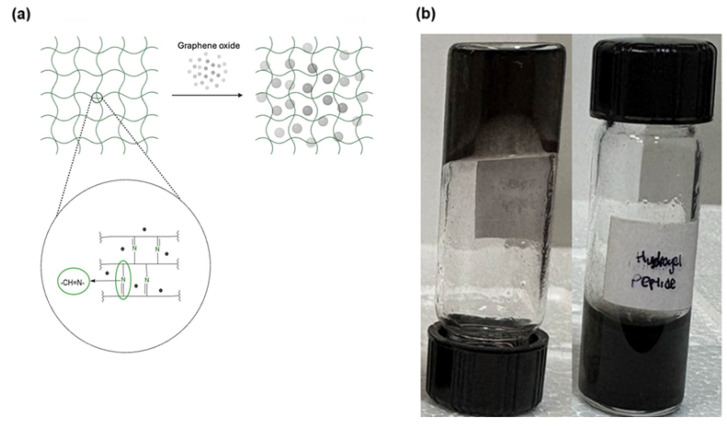
(**a**) Schematic description of the synthetic pathway for the preparation of the hydrogel composite of oxidized alginate (OA)/*N*-succinyl chitosan (NSC) and graphene oxide (GOx); (**b**) Picturee of the OA/NSC/GOx hydrogel composite.

The hydrogel was synthesized by cross-linking via a Schiff base reaction (Scheme 6). To do this, 0.15 g of dry oxidized alginate was dissolved in 5 mL of phosphate buffer solution (PBS, pH = 7.4) at room temperature. In parallel, 0.15 g of dry *N*-succinyl-chitosan was dissolved in 5 mL of the same phosphate buffer solution using an overhead mechanical stirrer; after 5–8 min of gentle stirring, NCS dissolution was complete. The oxidized alginate solution was added slowly, under stirring, to the *N*-succinyl-chitosan solution. The resulting mixture was heated to 37 °C, stirring until the formation of the hydrogel was observed. The final concentration obtained was 1.5% (*w*/*v*). Finally, 0.2 g of GOx or cRGD-GOx were added with stirring to reach a concentration of 0.2%, achieving the desired hydrogel composite reinforced with GOx or cRGD-GOx, depending on what product was required (Scheme 7). Nearly 2.5 mL of black hydrogel was obtained. The GOx/cRGD-GOx reinforced hydrogel composite was kept in refrigeration (−4 °C) until further use or lyophilized if required. Even at room temperature, the hydrogel was stable, and no signal of any transition that could be linked to the melting of the composite was observed.

### 3.8. Structural Characterizations

Samples underwent characterization using several analytical techniques. Fourier transform infrared spectroscopy (FTIR) was performed on an Agilent Technologies (Santa Clara, CA, USA) Cary 630 spectrophotometer with a diamond ATR detector, collecting 32 scans at a 4 cm^−1^ resolution across the 4000–650 cm^−1^ region. Raman scattering spectra were obtained using a Horiba Scientific (Irvine, CA, USA) XPlora-Plus Raman microscope with a 523 nm green laser and an automated platform. Morphological and chemical analyses were obtained with a high-resolution scanning field-emission electron microscope (HR-FESEM) (Tescan, MAIA 3, Brno, Czech Republic) operating at 10 keV with a Bruker Xflash 6|30 (Bremen, Germany) Energy Dispersive X-ray detector. TEM-EDS imaging was conducted on a FEI Tecnai G2 F20 FE-TEM (Hillsboro, OR, USA) at a 200 kV acceleration voltage with an Oxford Aztec (Concord, MA, USA) 80 mm SDD detector. Thermal behavior was assessed via thermogravimetric analysis (TGA) using a Netzsch STA 2500 Regulus (Selb, Germany) under N_2_ atmosphere, while differential scanning analysis (DSC) of 0.132 mg of the hydrogel composite was performed on a TA Instruments DSC-Model Q200 (New Castle, DE, USA) at 5 °C/min heating rate with N_2_ flow between 20 and 80 °C.

### 3.9. Injectability Procedure

The process was conducted using a sterile disposable hypodermic syringe (pink 18G × 25 mm needle), injecting 0.3 mL of hydrogel into the cardiac tissue of a chicken heart (7–9 weeks old) obtained from a local supplier. The heart was selected only if it looked healthy and without any physical damage. The injected tissue was then incubated in simulated body fluid (SBF) [97] at 37 °C and 1500 rpm for 12 h to simulate physiological conditions and ensure product retention. A longitudinal cut of the tissue was made to identify the injection site, followed by exposure to a constant water stream to confirm hydrogel adhesion to the tissue. Finally, a sample was observed under a microscope to evaluate the distribution and stability of the injected material, validating its ability to remain intact under simulated biological conditions.

### 3.10. Cell Culture of U937 Differentiated to Macrophages

The U937 cell line, derived from human diffuse histiocytic lymphoma and exhibiting monocytic characteristics, was cultured in Gibco-formulated RPMI-1640 medium, supplemented with 10% fetal bovine serum (FBS) for complete growth medium. A total of 1.5 million U937 cells were seeded and allowed to stabilize for 24 h under standard conditions (37 °C, 5% CO_2_). Macrophage differentiation was induced by treatment with 10 ng/mL PMA (phorbol-12-myristate-13-acetate) for 24 h, which promotes adhesion, morphological changes, and the expression of macrophage-specific surface markers [98,99]. After differentiation, cells were trypsinized, counted, and plated in 96-well plates at a density of 2000 cells/well. Differentiated macrophages were exposed to hydrogel for 24 h. TNF-α expression was quantified using a commercial ELISA kit (Invitrogen, Waltham, MA, USA; Cat. No. KHC3011), following the manufacturer’s protocol.

### 3.11. Cell Cytotoxicity and Proliferation Test

WS1 fibroblasts isolated from the skin of a Black female donor were cultured in ATCC-formulated Eagle’s Minimum Essential Medium (Catalog No. 30-2003), supplemented with fetal bovine serum (FBS) to a final concentration of 10%. To evaluate the cell viability of the gel, 3-[4,5-dimethylthiazol-2-yl]-2,5-diphenyltetrazolium bromide (MTT) and dimethyl sulfoxide (DMSO) were used. Initially, the cell culture wells were examined to identify possible morphological changes and verify the absence of contamination, with photographs taken as the first evidence of the cytotoxic effect. Subsequently, the MTT working solution was prepared, consisting of a mixture of 10% MTT, 7% FBS, and 10 µL/mL of antibiotic, with the volume adjusted using PBS. Additionally, three wells were included as reagent blank controls, to which only the MTT reagent was added. After removing the culture medium and washing each well with 100 µL of PBS, 37 µL of the MTT working solution was added, and the plates were incubated at 37 °C with 5% CO_2_ for 4 h with 5 µL of the synthesized hydrogel. At the end of the incubation period, 70 µL of DMSO was added, and the plates were stirred at 470 rpm for 15 min. Finally, absorbance readings were performed at 550 nm with a 620 nm reference filter, thus obtaining the necessary data for cell viability analysis.

### 3.12. Evaluation of Macrophage and Hydrogel Composite Interaction

Macrophage cell culture (3000 cells/well) and 150 mL of the hydrogel composite were incubated with 5 mL of RPMI 1640 medium at 37 °C with 5% CO_2_ for 4 h. After removing the culture medium and washing each well with 100 µL of PBS, cells were fixed with 4% fresh formaldehyde, incubated for 15 min at room temperature, and washed with 100 µL of PBS. Finally, the morphological assessment of the cells, stained with hematoxylin/eosin, was performed by optical microscopy (CX33; Olympus Live Science, Tokyo, Japan) with 40× and 100× lens (UPLXAPO20X; Olympus Live Science).

### 3.13. Rheology Study

The rheological properties were measured using a Kinexus Lab rheometer (Selb, Germany), equipped with a 20 mm diameter parallel plate geometry, with data analysis performed using OriginPro (Version 2024, OriginLab Corporation, Northampton, MA, USA). The OA-NSC/GOx hydrogel sample was applied to the 20 mm parallel plate and covered with silicone oil to prevent solvent evaporation. Measurement parameters included a dynamic frequency scan range of 0.1–100 rad s^−1^, stress amplitude of 0.1%, and a constant temperature of 25 °C.

## 4. Conclusions

A peptide-modified hydrogel composite was successfully engineered by integrating oxidized alginate (OA), *N*-succinyl-chitosan (NSC), and graphene oxide (GOx) functionalized with cyclic RGD peptide (c-RGD-GOx), demonstrating exceptional rheological properties for injectable applications, including shear-thinning behavior (viscosity reduction from 10 to <1 Pa·s) and elastic-dominated mechanics (G′ > G″), ensuring precise delivery while preserving structural stability. The c-RGD peptide enhanced bioactivity, evidenced by stable covalent integration (EDS-confirmed nitrogen mapping) and robust fibroblast viability (≥95%, MTT assay), while significantly suppressing TNF-α secretion in macrophages by 80% (30 vs. 150 pg/μL in controls), underscoring anti-inflammatory potential. The hydrogel’s interconnected porous architecture (50–200 μm pore size, SEM) mimics native extracellular matrix topology, facilitating cell infiltration and nutrient exchange, with adhesion tests in chicken cardiac tissue confirming retention under dynamic conditions. Despite these advancements, limitations include the absence of in vivo validation, untested mechanical resilience under physiological stress (e.g., cyclic cardiac strain), and unaddressed scalability for clinical translation. Future studies must prioritize preclinical evaluation in disease models, functionalization with angiogenesis-promoting or anti-fibrotic agents, dynamic mechanical testing, and scalability protocols addressing sterilization and long-term stability. By bridging minimally invasive delivery with bioactive complexity, this hydrogel represents a transformative leap in regenerative medicine. Realizing its full potential, however, demands interdisciplinary collaboration across materials science, biology, and clinical research to overcome translational barriers and optimize therapeutic outcomes for complex tissue defects. In summary, this composite hydrogel represents a multifunctional advancement over conventional systems, combining injectability, targeted bioactivity, and anti-inflammatory properties, with potential for applications in cardiac regeneration, complex wounds, and beyond.

## Data Availability

Data is contained in the paper and Supplementary Materials.

## Supplementary Materials

The following supporting information can be downloaded at https://www.mdpi.com/article/10.3390/ph18050616/s1. TGA curves for OA, NSC, GOx and OA/NSC/GOx hydrogels; 2D EDS mapping for OA/NSC/GOx hydrogel composite; and product information, as provided by supplier, for alginate and chitosan.

## Abbreviations

The following abbreviations are used in this manuscript:

c-RGD	Cyclic arginylglycylaspartic acid peptide
ECM	Extracelular matrix
TGA	Thermogravimetric analysis
FTIR	Fourier transform infrared
SEM	Scanning electron microscopy
EDS	Energy dispersive spectroscopy
TEM	Transmission electron microscopy
GOx	Graphene oxide
OA	Oxidized alginate
NSC	*N*-succinyl chitosan
ELISA	Enzyme-linked immunosorbent assay
TNF-α	Tumor necrosis factor alpha
MTT	3-[4,5-dimethylthiazol-2-yl]-2,5 diphenyl tetrazolium bromide
PMA	Phorbol-12-myristate-13-acetate
DIC	*N*,*N*′-Diisopropylcarbodiimide
TFA	Trifluoroacetic acid
HPLC	High performance liquid chromatography
DMF	Dimethylformamide
TIS	Triisopropylsilane
MWCO	Molecular weight cut-off
NHS	*N*-hydroxysuccinamide
EDC	1-Ethyl-3-(3-dimethylaminopropyl)-carbodiimide
ATR	Attenuated total reflectance
PBS	Phosphate buffer solution
DMSO	Dimethylsulfoxide
ATCC	American Type Culture Collection
