# Supplementary material for: Development and Characterization of an Injectable Alginate/Chitosan Composite Hydrogel Reinforced with Cyclic-RGD Functionalized Graphene Oxide for Potential Tissue Regeneration Applications
Source: Pharmaceuticals (Basel). 2025 Apr 23;18(5):616. doi: 10.3390/ph18050616 (PMC12115116; doi:10.3390/ph18050616)
Supplement: Supplementary file 1 [file pharmaceuticals-18-00616-s001.zip › pharmaceuticals-3569744-supplementary.pdf]

Supplementary information

Development and Characterization of an Injectable Alginate/Chitosan Composite Hydrogel Reinforced with Cyclic-RGD Functionalized Graphene Oxide for Potential Tissue Regeneration Applications

Mildred A. Sauce-Guevara 1, Sergio D. García-Schejtman 2, Emilio I. Alarcon 2,3, Sergio A. Bernal-Chavez 1,* and Miguel A. Mendez-Rojas 1,*

1 Department of Chemical and Biological Sciences, Universidad de las Americas Puebla, Ex-Hacienda de Santa Catarina Martir s/n, San Andres Cholula, Puebla 72820, Mexico

2 Bioengineering and Therapeutic Solutions (BEaTS) Program, University of Ottawa Heart Institute, Ottawa, ON K1Y 4W7, Canada

3 Department of Biochemistry, Microbiology, and Immunology, University of Ottawa, Ottawa, ON K1H 8M5, Canada

* Correspondence: sergio.bernal@udlap.mx (S.A.B.-C.); miguela.mendez@udlap.mx (M.A.M.-R.) ; Tel.: +52-222-2292607 (M.A.M.-R.)

TGA Analysis

Comparatives TGA curves for: a) alginate and oxidized alginate (OA); b) chitosan and N-succinyl chitosan (NSC); (c) graphene oxide (GOx) and OA/NSC/GOx hydrogel composite

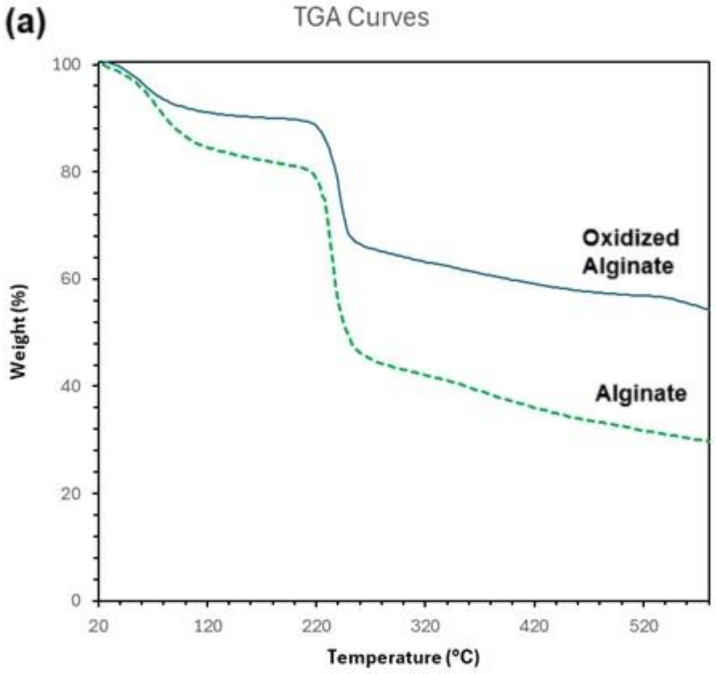

(b)

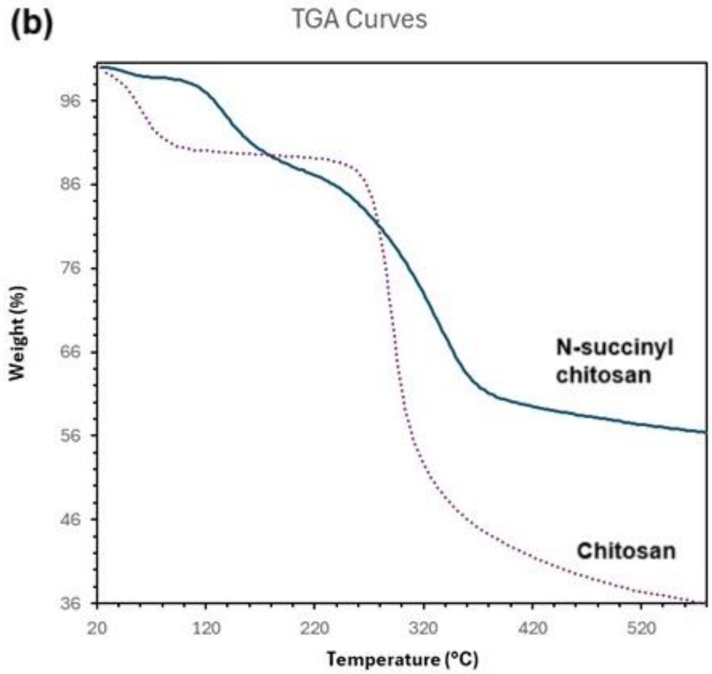
TGA Curves
Weight (%)
96
86
76
66
56
46
36
20
120
220
320
420
520
Temperature (°C)
N-succinyl
chitosan
Chitosan

(c)

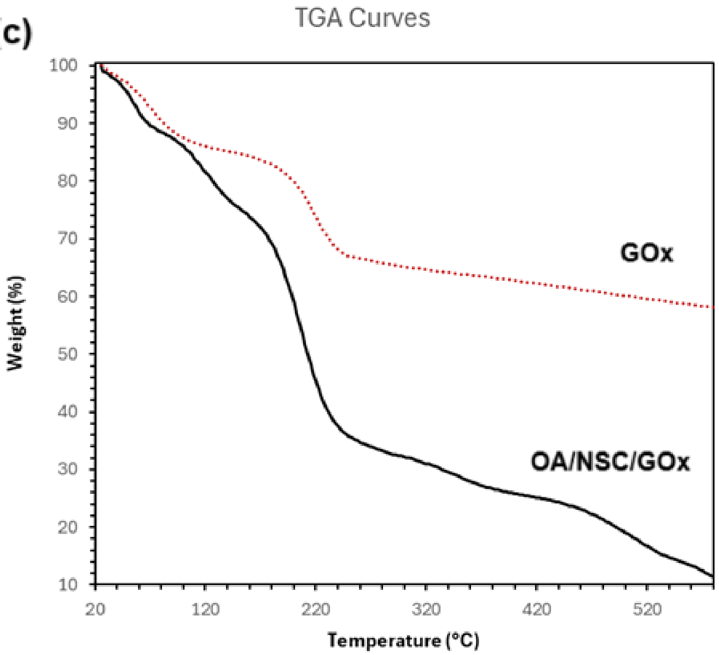
TGA Curves
Weight (%)
100
90
80
70
60
50
40
30
20
10
20
120
220
320
420
520
Temperature (°C)
GOx
OA/NSC/GOx

2D EDS mapping of the OA/NSC/GOx hydrogel composite

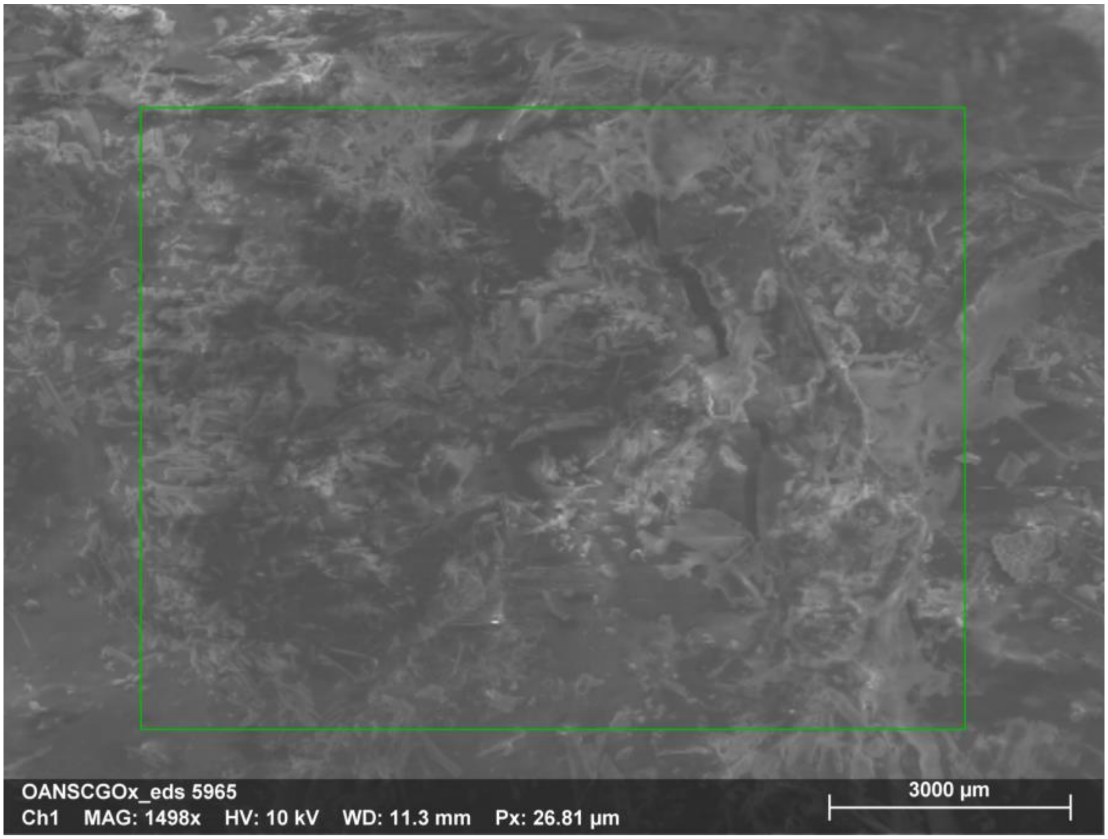

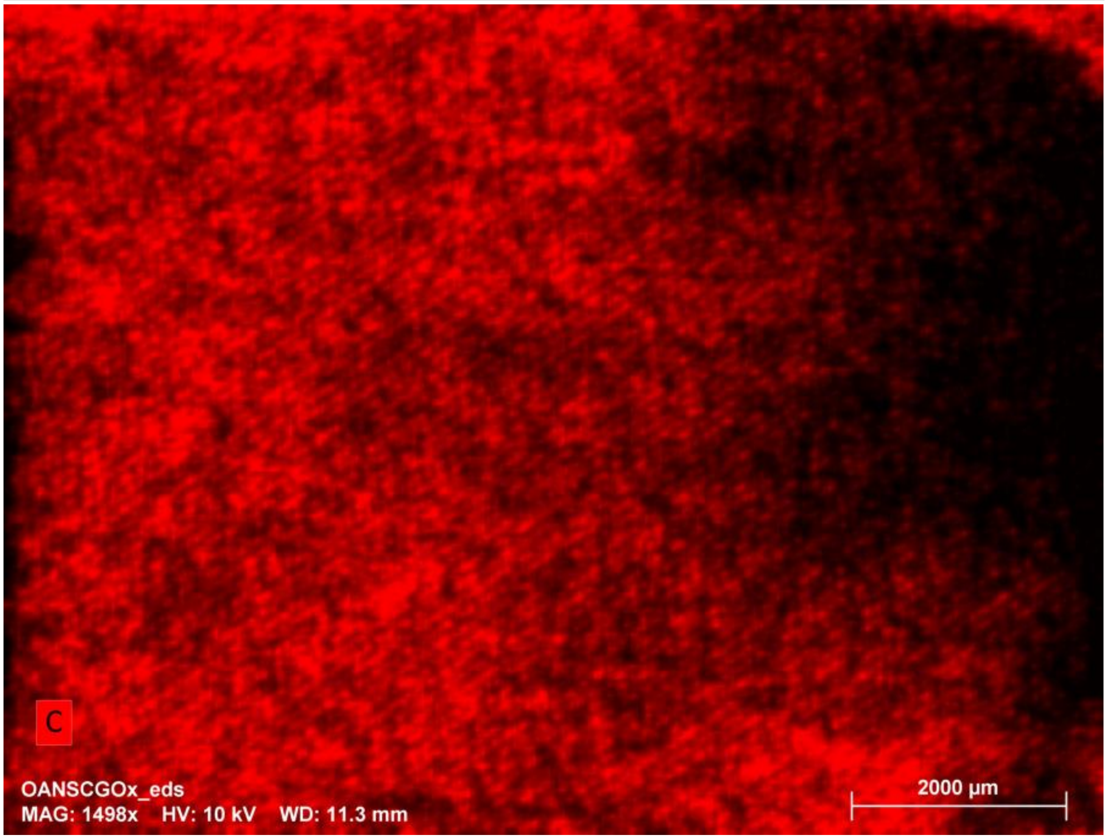

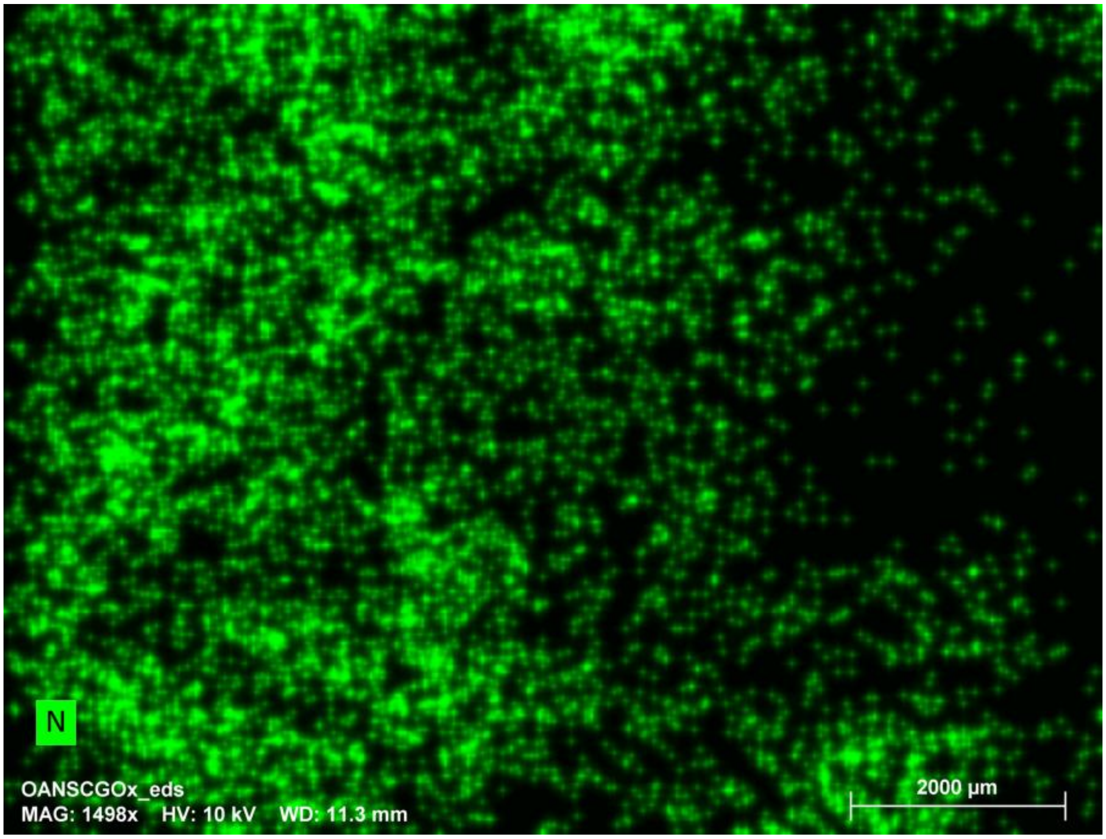
N
OANSCGOx_eds
MAG: 1498x HV: 10 kV WD: 11.3 mm
2000 µm

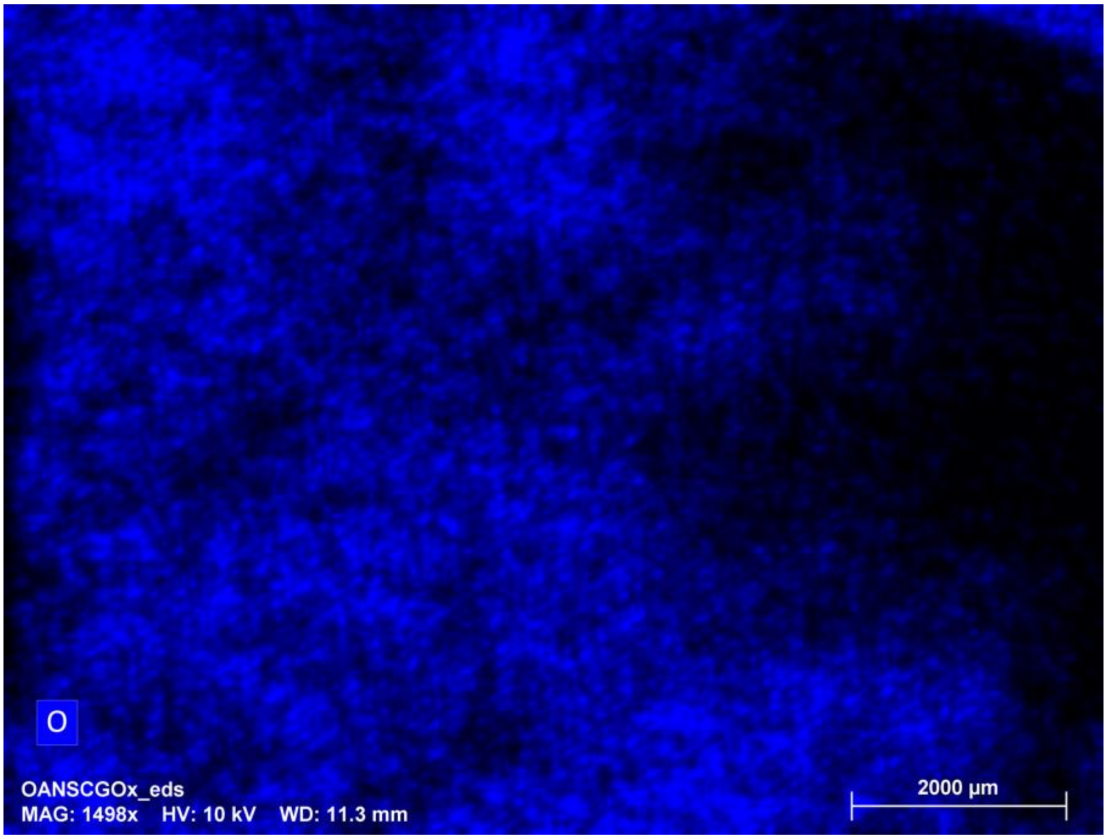
O
OANSCGOx_eds
MAG: 1498x HV: 10 kV WD: 11.3 mm
2000 µm

Product Information for sodium alginate (as provided by the supplier):

SIGMA-ALDRICH

sigma-aldrich.com

3050 Spruce Street, Saint Louis, MO 63103, USA
Website: www.sigmaaldrich.com
Email USA: techserv@sial.com
Outside USA: eurtechserv@sial.com

Product Specification

Product Name:
Alginic acid sodium salt from brown algae – Medium viscosity

Product Number: **A2033**
CAS Number: 9005-38-3
MDL: MFCD00081310

Storage Temperature: 2 - 8 ℃

TEST	Specification
Appearance (Color)	White to Beige and Faint Brown to Brown
Appearance (Form)	Powder
Solubility (Color)	Faint Yellow to Yellow
Solubility (Turbidity)<br>100 mg plus 10 mL of Water	Slightly Hazy to Very Hazy
Brookfield Viscosity<br>2% in water at 25 Deg C	≥ 2000 cps
Recommended Retest Period<br>2 Years	------------------------

Specification: PRD.1.ZQ5.10000009292

Sigma-Aldrich warrants, that at the time of the quality release or subsequent retest date this product conformed to the information contained in this publication. The current Specification sheet may be available at Sigma-Aldrich.com. For further inquiries, please contact Technical Service. Purchaser must determine the suitability of the product for its particular use. See reverse side of invoice or packing slip for additional terms and conditions of sale.

Product Information for chitosan (as provided by the supplier):

Sigma-Aldrich.

3050 Spruce Street, Saint Louis, MO 63103, USA
Website: www.sigmaaldrich.com
Email USA: techserv@sial.com
Outside USA: eurtechserv@sial.com

Product Specification

Product Name:
Chitosan - medium molecular weight

Product Number: **448877**
CAS Number: 9012-76-4
MDL: MFCD00161512
Formula: C12H24N2O9

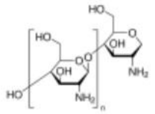

TEST	Specification
Appearance (Color)	Off White to Beige and Faint Brown to Light Brown
Appearance (Form)<br>Powder and/or Chips	Conforms to Requirements
Deacetylation	$\geq$ 75 %
Viscosity<br>c = 1%, 1% Acetic Acid	200 - 800 cps

Specification: PRD.2.ZQ5.10000030084

Sigma-Aldrich warrants, that at the time of the quality release or subsequent retest date this product conformed to the information contained in this publication. The current Specification sheet may be available at Sigma-Aldrich.com. For further inquiries, please contact Technical Service. Purchaser must determine the suitability of the product for its particular use. See reverse side of invoice or packing slip for additional terms and conditions of sale.
